# Supplementary material for: NK Cells Acquire CCR5 and CXCR4 by Trogocytosis in People Living with HIV-1
Source: Vaccines (Basel). 2022 Apr 28;10(5):688. doi: 10.3390/vaccines10050688 (PMC9145773; doi:10.3390/vaccines10050688)
Supplement: Supplementary file 1 [file vaccines-10-00688-s001.zip › vaccines-1660525-supplementary.pdf]

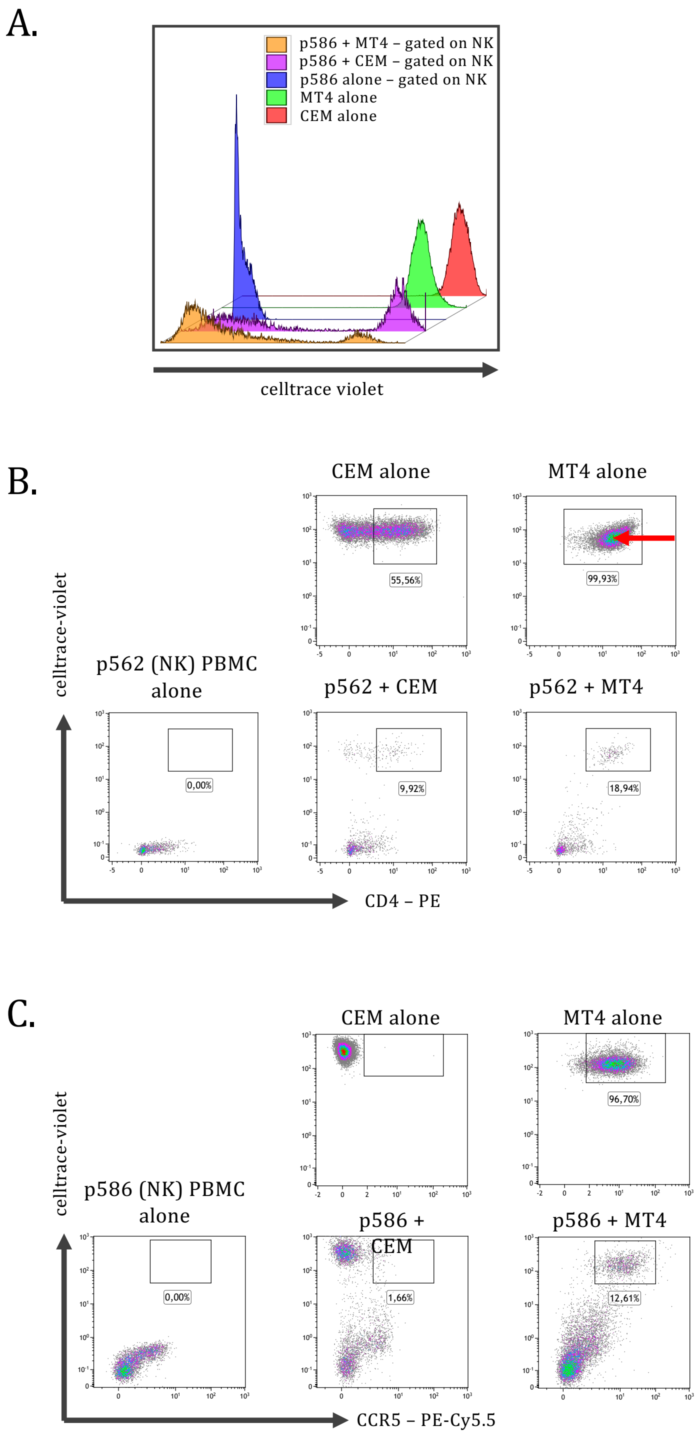

Fig. S1 NK cells from healthy donors uptake molecules from T cells. A) NK cells were incubated at 1:5 [E:T] ratio for 4 h with MT4 and CEM T cell lines that had been labeled with celltrace violet (CTV). Transfer of HIV receptor CD4 (B) and CCR5 (C) from target cells to NK cells.

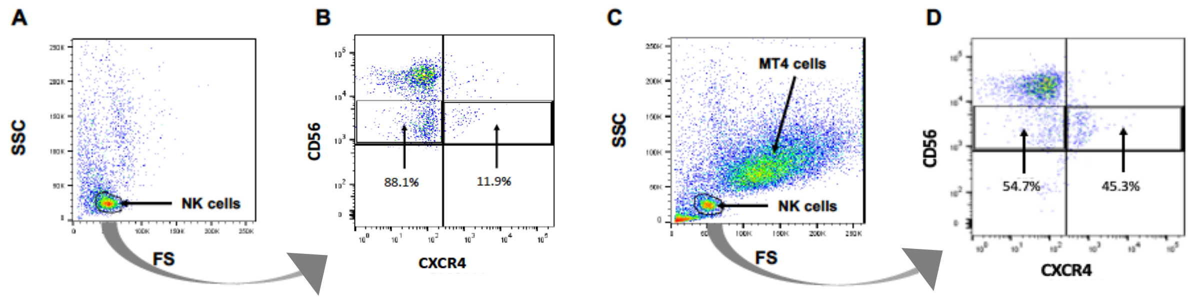

Figure S2. Primary expanded NK cells (eNK) acquire CXCR4 cell surface expression upon coculture with CXCR4-positive MT4 cells. (A) Size and granulometry of eNK. (B) CXCR4 expression at the surface of eNK cultured alone. (C) Size and granulometry of eNK cocultured with MT4 cells. (D) CXCR4 expression at the surface of eNK cocultured with MT4 cells.

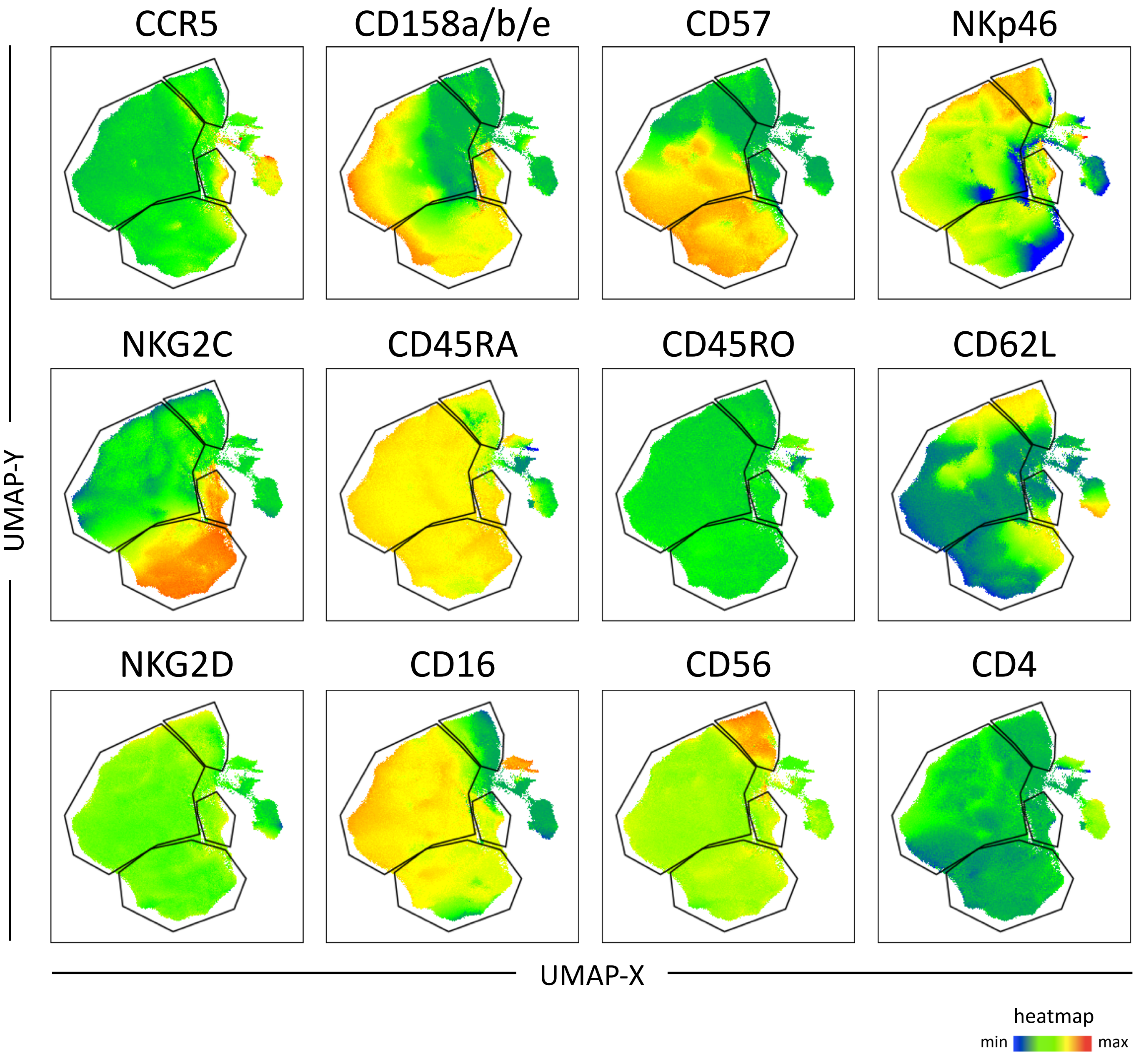

Figure S3. Heatmaps of individual markers that constitute the UMAP in Figure 3.

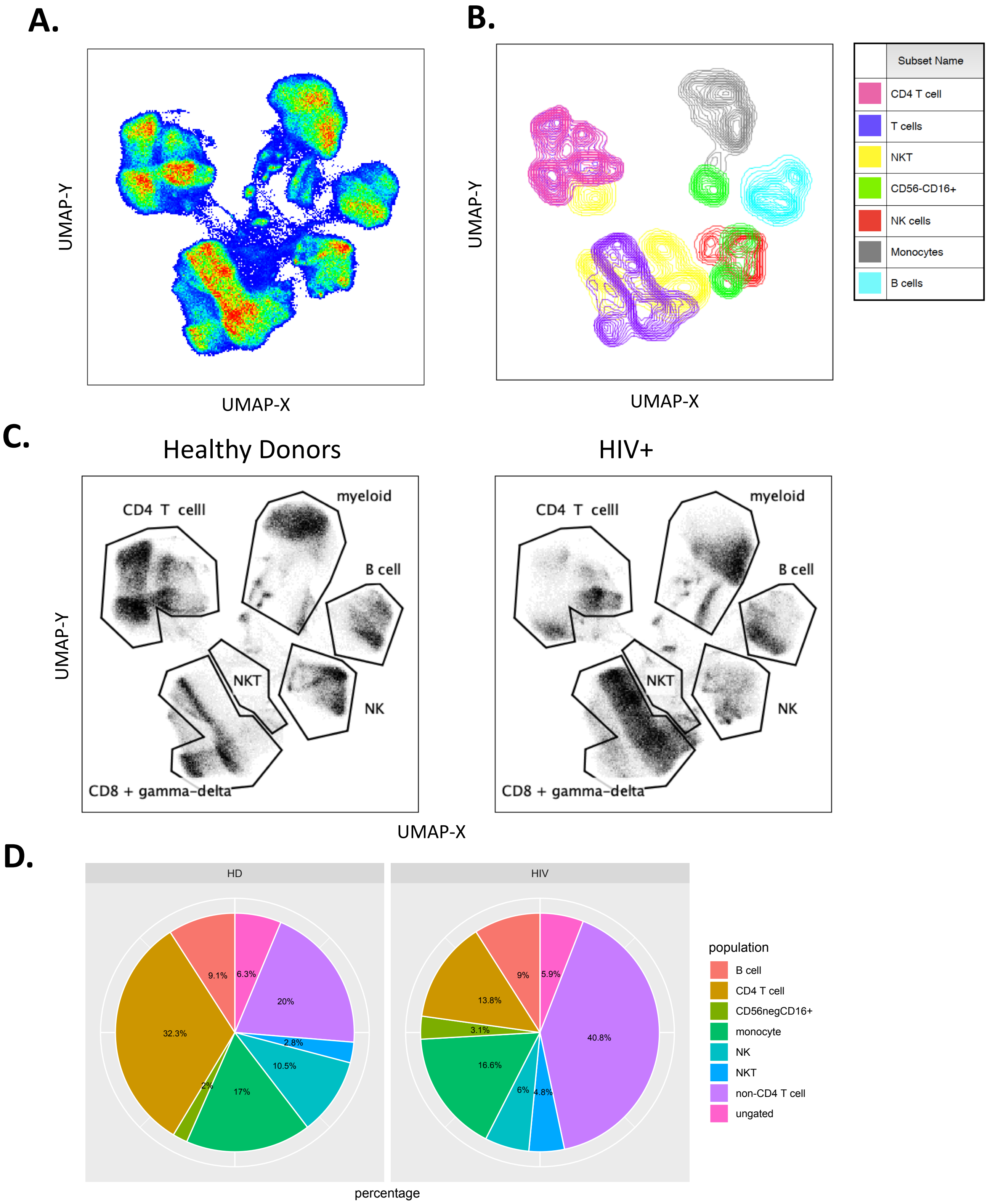

Figure S4. UMAP (Uniform Manifold Approximation and Projection for Dimension Reduction) analysis of 50'000 viable cells of healthy donors and HIV+ patients and constitution of PBMC populations. A) General UMAP constituted from all sample (HD: n=4, HIV: n=4). B) Overlay projection of each PBMC population on the general UMAP in panel A, using manual gating strategy. C) PBMC immune populations on UMAP derived from each group. D) Pie charts of quantified percentage of PBMC clusters between HD and HIV as shown in panel C.

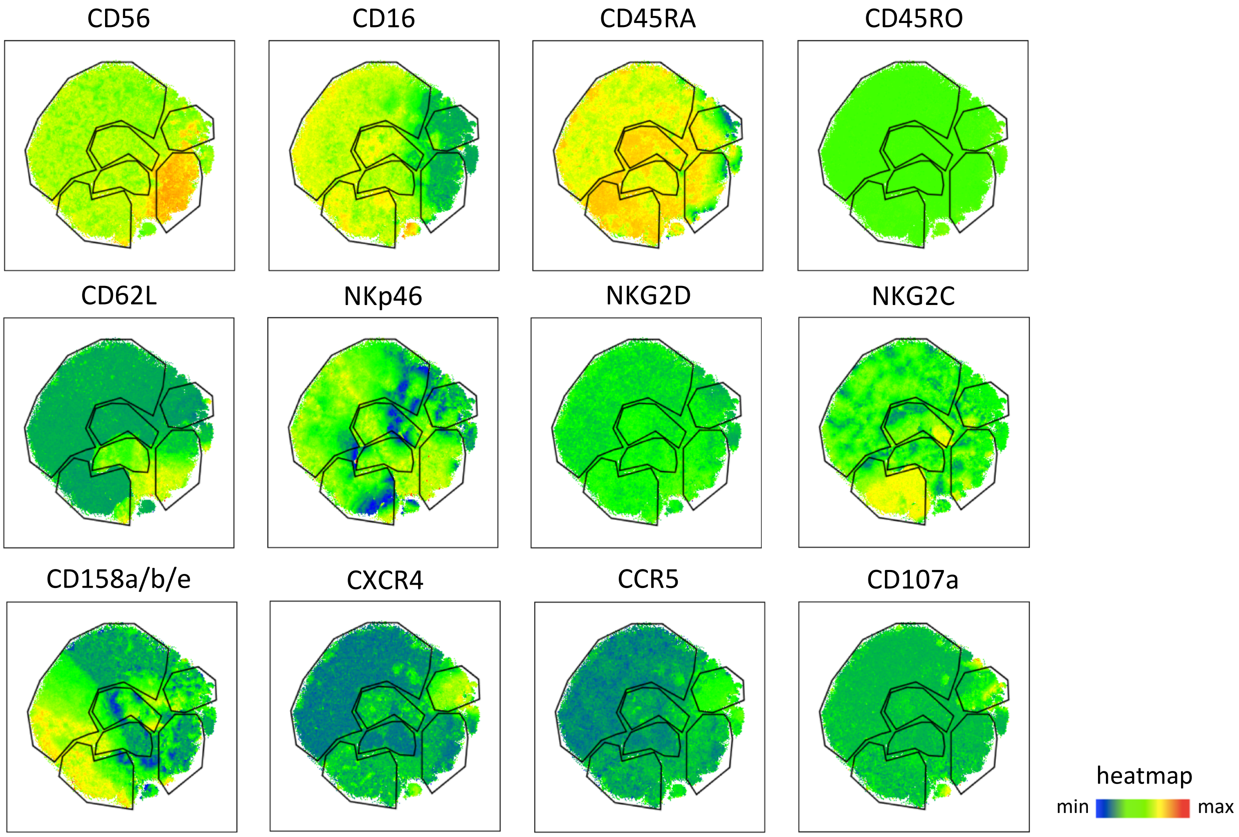

Figure S5. Heatmaps of the expression of the 12 surface markers used to constitute the original UMAP embed on Figure 7.

C.

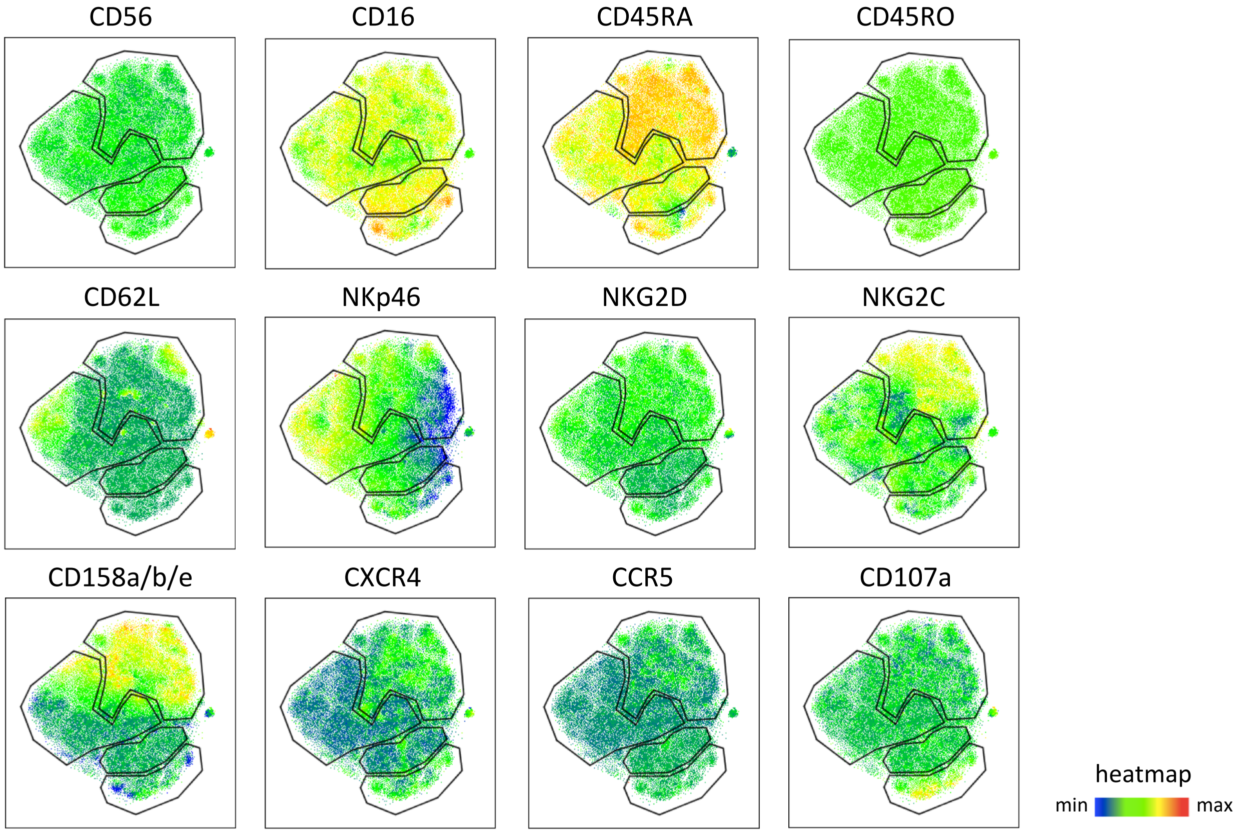

Figure S6. Heatmaps of the expression of the 12 surface markers used to constitute the original UMAP embed on Figure 8.

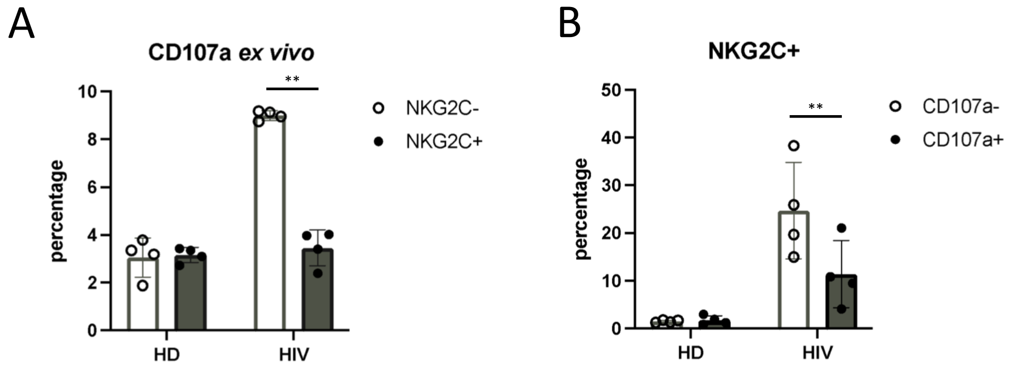

Figure S7. Expanded NKG2C+ NK population in viremic HIV patients did not correlate with enhanced in vivo degranulation. A) Level of CD107a detected ex vivo in NK cells from HD and HIV group stratified by NKG2C status. B) Percentage of NKG2C+ NK cells found in CD107a+ or in CD107a- cells from HD or HIV groups. Statistical significance was determined by paired t-test; ** $p \leq 0.01$.
